# Supplementary material for: HSP90-CDC37-PP5 forms a structural platform for kinase dephosphorylation
Source: Nat Commun. 2022 Nov 29;13:7343. doi: 10.1038/s41467-022-35143-2 (PMC9709061; doi:10.1038/s41467-022-35143-2)
Supplement: Supplementary file 1 — Supplementary Information [file 41467_2022_35143_MOESM1_ESM.pdf]

SUPPLEMENTARY FIGURE 1

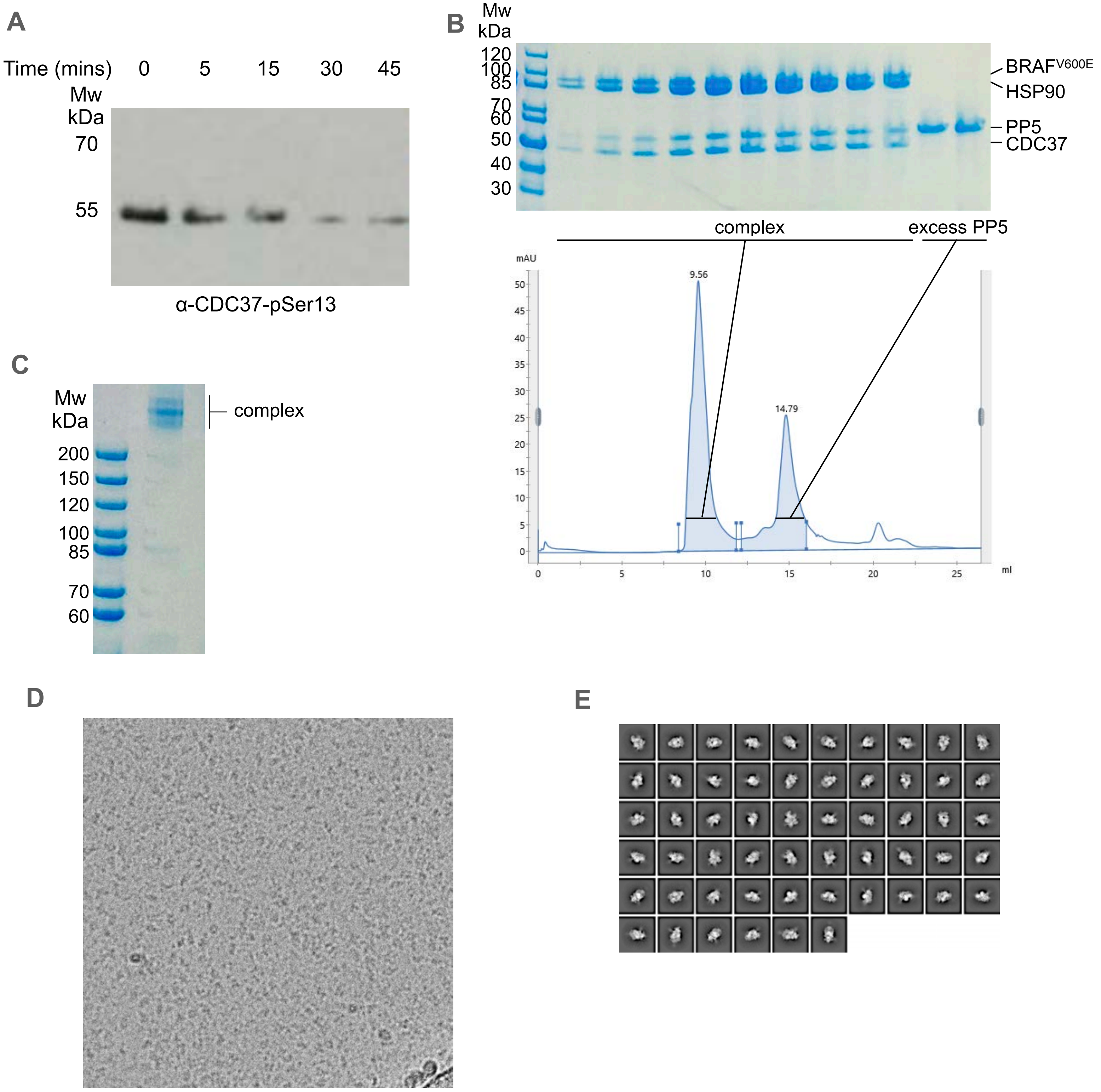

PP5 activity and complex assembly

A: Western blot developed with phosphospecific antibody to pSer13 showing time course of dephosphorylation of CDC37-pSer13 when PP5 is added to an HSP90-CDC37-BRAF^{V600E} complex. Image shown is representative of > 3 independent repeats **B:** Coomassie-stained SDS PAGE gel showing purification of HSP90-CDC37-BRAF^{V600E}-PP5 complex by size exclusion chromatography. Elution profile from S200 is shown below. **C:** Coomassie-stained SDS PAGE gel showing high molecular weight complex formed by BS3 cross linking of peak fractions from **B. D:** Example electron micrograph of cryogenically preserved sample of C applied to grids. **E**: 2D class averages of particles picked from micrographs, with inverted contrast. Clear secondary structure elements are visible at this stage.

SUPPLEMENTARY FIGURE 2

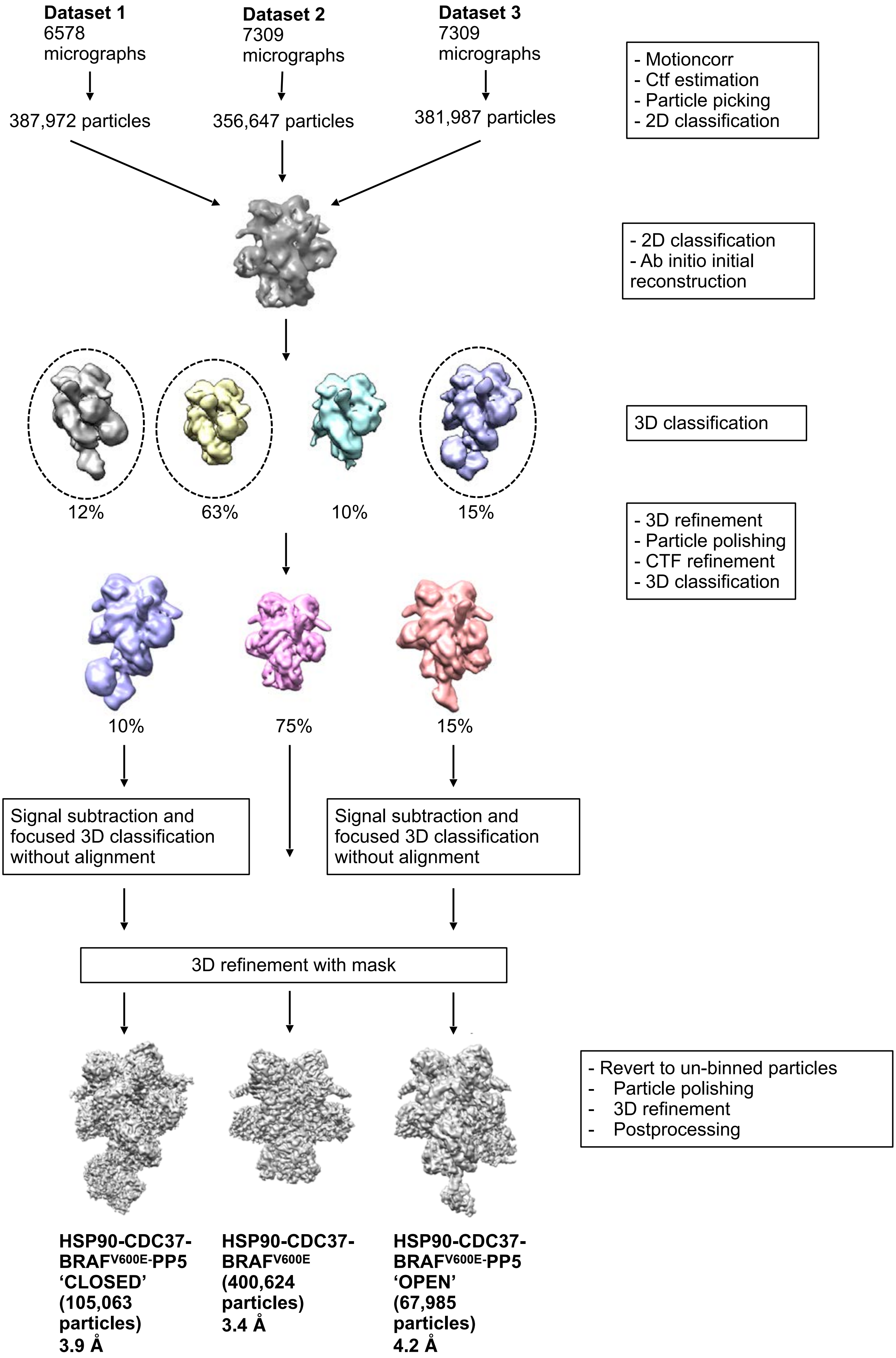

CryoEM processing schema

Stages in the image processing of the data used to generate the structures described here

SUPPLEMENTARY FIGURE 3

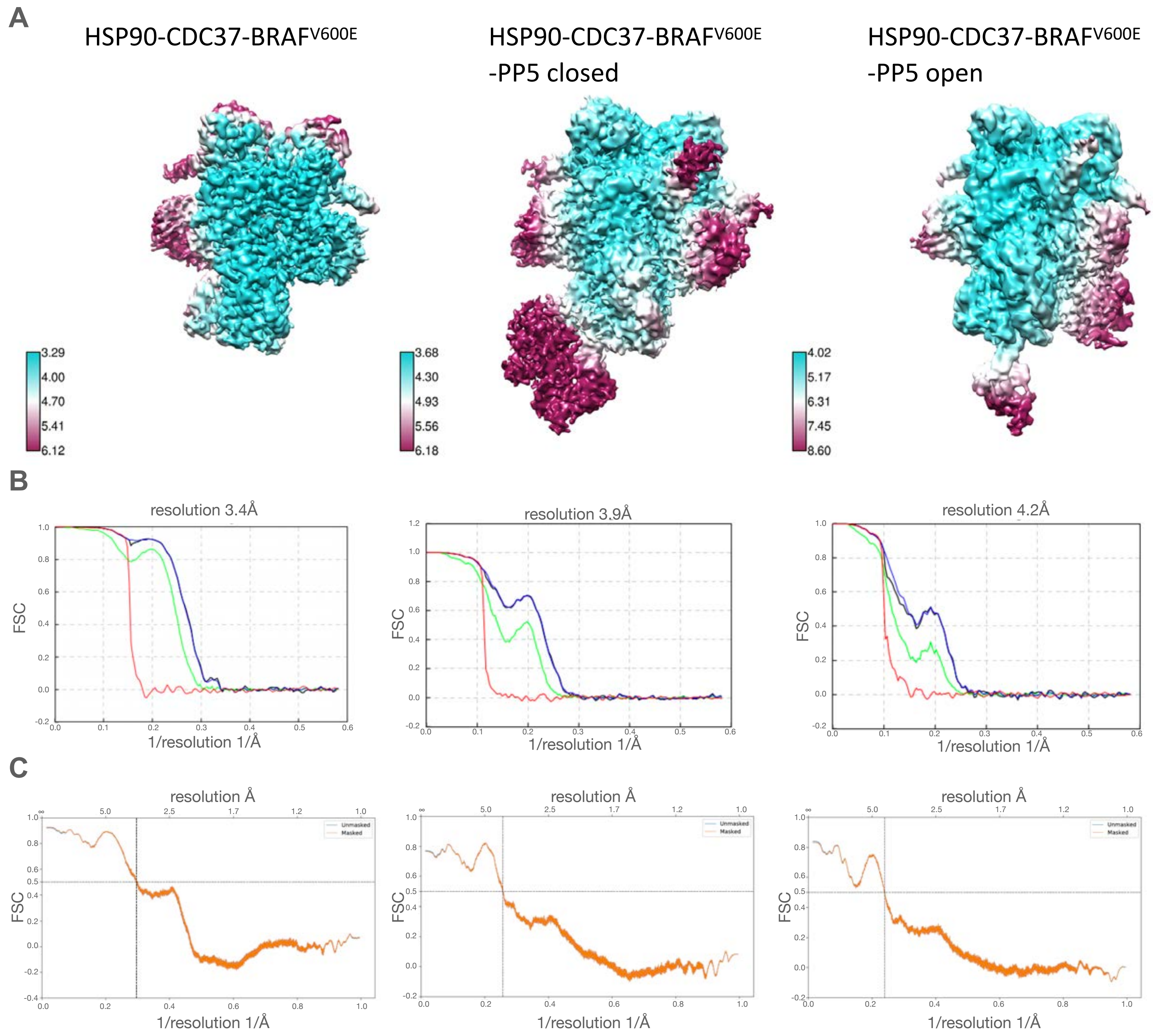

CryoEM resolution analysis

A: CryoEM volumes for the three structures reported here, surface coloured to reflect the local resolution estimated by RELION4.0 as shown in the colour keys. **B**: Fourier Shell Correlation plots (RELION 4.0) for the three structures reported here. Black curve = corrected, green = unmasked, blue = masked, phase randomised masked. **C**:Map-Model FSC plots for the three structures reported here - calculated using there Mtriage function of phenix

SUPPLEMENTARY FIGURE 4

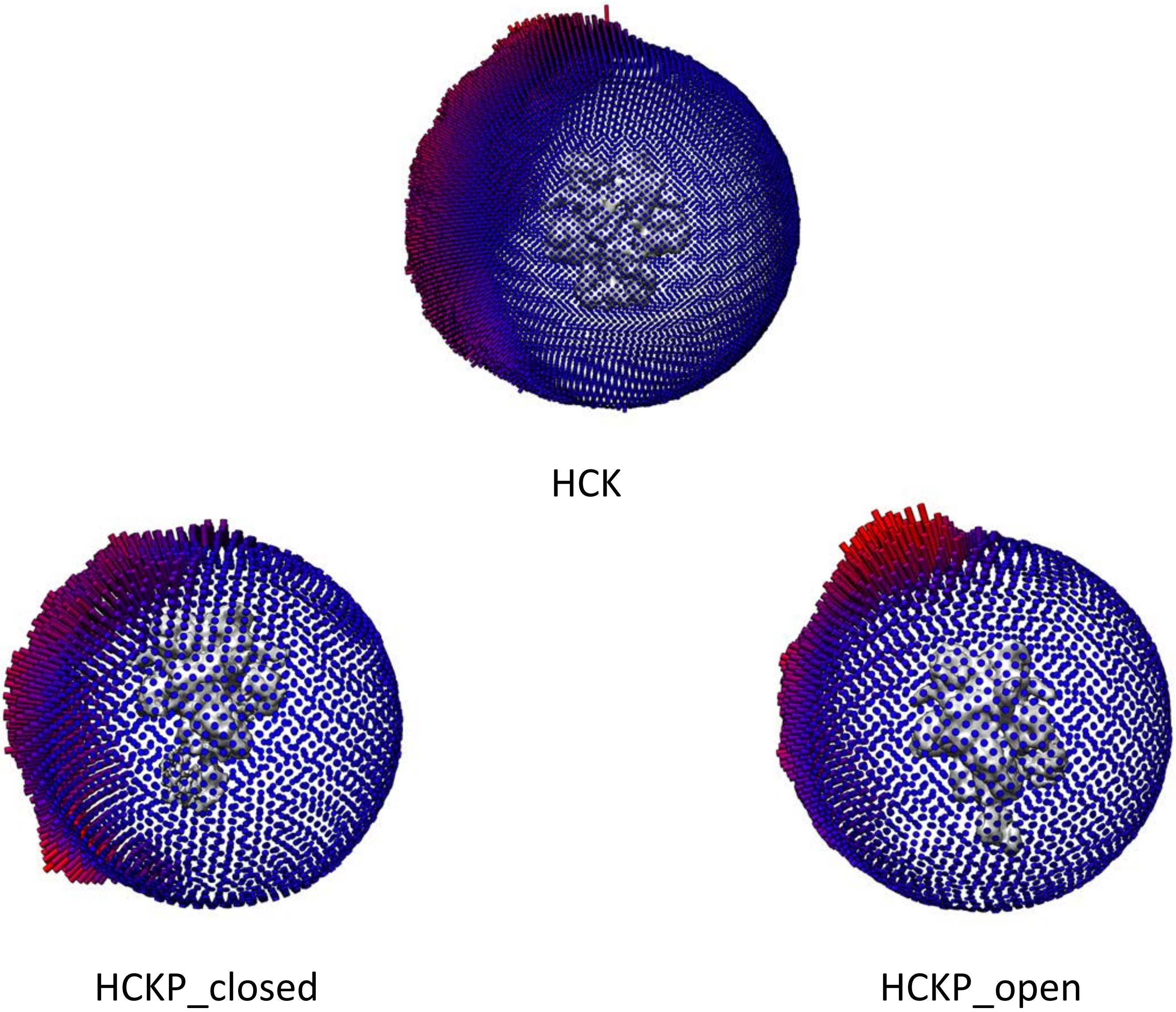

CryoEM particle orientation analysis

3D plots of distribution of particle orientations for the three structures presented here, calculated in RELION4. The degree of projection of the dot from the surface of the circle is proportional to the frequency of observation, with the colour ramping from blue for the least frequent to red for the most frequent. Essentially all possible views are represented for all structures, with views orthogonal to the widest presentation of the particle more highly represented.

SUPPLEMENTARY FIGURE 5

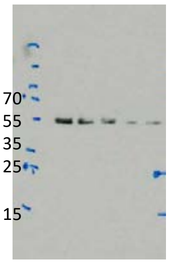

α pSer13
Suppl Fig. 1

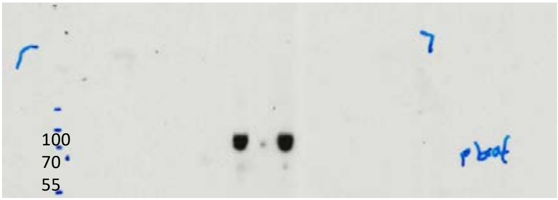

α pSer729 BRAF - Figure 5B

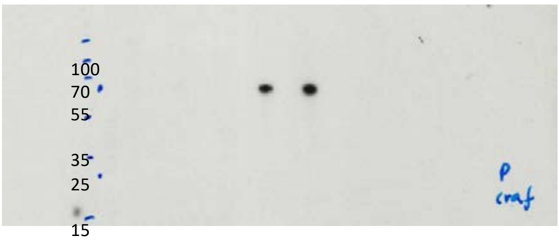

α pSer621 CRAF – Figure 5D

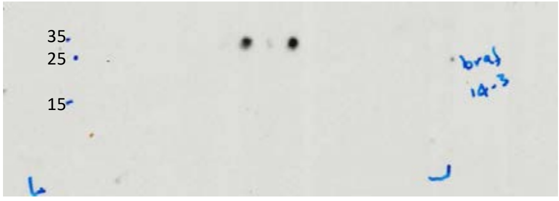

α 14-3-3 (BRAF complex binding) – Figure 5B

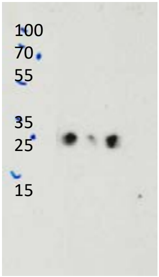

α 14-3-3 (CRAF complex binding) – Figure 5D

Uncropped western blots

The original uncropped images of the various western blots presented in the main figures as indicated.
