# Supplementary material for: HSP90-CDC37-PP5 forms a structural platform for kinase dephosphorylation
Source: Nat Commun. 2022 Nov 29;13:7343. doi: 10.1038/s41467-022-35143-2 (PMC9709061; doi:10.1038/s41467-022-35143-2)
Supplement: Supplementary file 3 — Description of Supplementary Files [file 41467_2022_35143_MOESM3_ESM.docx]

**File Name SUPPLEMENTARY MOVIE 1.**

**Description : Opening and Closing of PP5**

Following binding to either of the two symmetry equivalent binding sites at the C-terminus of HSP90 the phosphatase domain of PP5 can release from its autoinhibited interaction with its own TPR domain, and engage with substrate phosphorylation sites on the chaperone components or the bound client. The movie shows the transition between the experimentally determined ‘closed’ and ‘open’ structures, with the PP5 component of the ‘closed’ structure rotated to the same side as the phosphatase in the experimental ‘open’ structure by superimposition of the TPR domains.
