# Supplementary material for: HSP90-CDC37-PP5 forms a structural platform for kinase dephosphorylation
Source: Nat Commun. 2022 Nov 29;13:7343. doi: 10.1038/s41467-022-35143-2 (PMC9709061; doi:10.1038/s41467-022-35143-2)
Supplement: Supplementary file 5 — Reporting Summary [file 41467_2022_35143_MOESM5_ESM.pdf]

nature portfolio

Corresponding author(s): Laurence Pearl

Last updated by author(s): Oct 19, 2022

Reporting Summary

Nature Portfolio wishes to improve the reproducibility of the work that we publish. This form provides structure for consistency and transparency in reporting. For further information on Nature Portfolio policies, see our Editorial Policies and the Editorial Policy Checklist.

Statistics

For all statistical analyses, confirm that the following items are present in the figure legend, table legend, main text, or Methods section.

n/a	Confirmed	
☐	☒	The exact sample size (n) for each experimental group/condition, given as a discrete number and unit of measurement
☒	☐	A statement on whether measurements were taken from distinct samples or whether the same sample was measured repeatedly
☐	☒	The statistical test(s) used AND whether they are one- or two-sided<br>*Only common tests should be described solely by name; describe more complex techniques in the Methods section.*
☒	☐	A description of all covariates tested
☒	☐	A description of any assumptions or corrections, such as tests of normality and adjustment for multiple comparisons
☒	☐	A full description of the statistical parameters including central tendency (e.g. means) or other basic estimates (e.g. regression coefficient) AND variation (e.g. standard deviation) or associated estimates of uncertainty (e.g. confidence intervals)
☒	☐	For null hypothesis testing, the test statistic (e.g. F, t, r) with confidence intervals, effect sizes, degrees of freedom and P value noted<br>*Give P values as exact values whenever suitable.*
☒	☐	For Bayesian analysis, information on the choice of priors and Markov chain Monte Carlo settings
☒	☐	For hierarchical and complex designs, identification of the appropriate level for tests and full reporting of outcomes
☒	☐	Estimates of effect sizes (e.g. Cohen's d, Pearson's r), indicating how they were calculated

Our web collection on statistics for biologists contains articles on many of the points above.

Software and code

Policy information about availability of computer code

Data collection	CryoEM data were collected with EPU version 2.11 (Thermo Fisher Scientific)<br>LC-MS analysis utilised an Orbitrap Lumos Mass Spectrometer (Thermo Fisher Scientific)
Data analysis	Electron micrographs were analysed using cryoSPARC2 v3.3.1 and RELION3.1 and RELION4. Model building utilised COOT 0.8.9.2-pre EL (revision count 7607).<br>Model refinement utilised PHENIX version 1.20.1-4487-000.<br>Mass spectrometry data were processed with Proteome Discoverer 2.4 (Thermo Fisher Scientific) and Perseus 1.6.2.2 https://www.nature.com/articles/nmeth.3901

For manuscripts utilizing custom algorithms or software that are central to the research but not yet described in published literature, software must be made available to editors and reviewers. We strongly encourage code deposition in a community repository (e.g. GitHub). See the Nature Portfolio guidelines for submitting code & software for further information.

Data

Policy information about availability of data

All manuscripts must include a data availability statement. This statement should provide the following information, where applicable:

- Accession codes, unique identifiers, or web links for publicly available datasets
- A description of any restrictions on data availability
- For clinical datasets or third party data, please ensure that the statement adheres to our policy

CryoEM models and maps have been deposited in PDB and EMD as follows : HCK - PDB ID 7ZR0, EMD-14875; HCKPo - PDB ID 7ZR6, EMD-14884; HCKPc PDB ID 7ZR5, EMD-14883.
Mass spectrometry data for HSP90-CDC37-BRAF and HSP90-CDC37-CRAF phosphorylation analyses have been deposited with ProteomeXchange with Project accession codes : PXD033678 and PXD035934 respectively

Human research participants

Policy information about studies involving human research participants and Sex and Gender in Research.

Reporting on sex and gender	N/A
Population characteristics	N/A
Recruitment	N/A
Ethics oversight	N/A

Note that full information on the approval of the study protocol must also be provided in the manuscript.

Field-specific reporting

Please select the one below that is the best fit for your research. If you are not sure, read the appropriate sections before making your selection.

☒ Life sciences ☐ Behavioural & social sciences ☐ Ecological, evolutionary & environmental sciences

For a reference copy of the document with all sections, see nature.com/documents/nr-reporting-summary-flat.pdf

Life sciences study design

All studies must disclose on these points even when the disclosure is negative.

Sample size	CryoEM structures were determined from the best data sets collected after extensive optimization to achieve op[timal particle density and ice thickness. Sample sizes were sufficient for the resolutions acheived
Data exclusions	For cryoEM all collected micrographs for which a CTF could be modelled, were included in the analysis. For mass spectrometry, only peptides with signal-to-noise ratio > 3 were used.
Replication	Mass spectrometry analysis used duplicate technical repeats. All repeats were successful.
Randomization	No randomization was applied to samples as the concept is meaningless for the type of analyses employed
Blinding	No blinding experiments were performed as the concept is meaningless for the type of analyses employed

Reporting for specific materials, systems and methods

We require information from authors about some types of materials, experimental systems and methods used in many studies. Here, indicate whether each material, system or method listed is relevant to your study. If you are not sure if a list item applies to your research, read the appropriate section before selecting a response.

Materials & experimental systems

n/a	Involved in the study
☐	☒ Antibodies
☐	☒ Eukaryotic cell lines
☒	☐ Palaeontology and archaeology
☒	☐ Animals and other organisms
☒	☐ Clinical data
☒	☐ Dual use research of concern

Methods

n/a	Involved in the study
☒	☐ ChIP-seq
☒	☐ Flow cytometry
☒	☐ MRI-based neuroimaging

Antibodies

Antibodies used	Recombinant Anti-BRAF (phospho S729) antibody [EPR2207] (ab124794) (Abcam); Recombinant Anti-Raf1 (phospho S621) antibody [EPR1521(2)] (ab157201); Anti-phospho-CDC37 (pSer13) antibody (MA533209) (Invitrogen); anti-14-3-3 antibody (#8312) Cell Signalling
Validation	Manufacturers validation against purified antigen, investigator verified by evident reactivity with purified recombinant material used in this study.

Eukaryotic cell lines

Policy information about cell lines and Sex and Gender in Research

Cell line source(s)	Protein expression utilised Spodoptera frugiperda Sf9 cells originally purchased from Invitrogen and maintained locally
Authentication	Cells support replication of baculovirus which is adequate authentication for protein expression
Mycoplasma contamination	Cells tested mycoplasma negative in regular routine testing during the course of the study
Commonly misidentified lines (See ICLAC register)	None
